# Development and validation of an endoscopic images-based deep learning model for detection with nasopharyngeal malignancies

**DOI:** 10.1186/s40880-018-0325-9

**Published:** 2018-09-25

**Authors:** Chaofeng Li, Bingzhong Jing, Liangru Ke, Bin Li, Weixiong Xia, Caisheng He, Chaonan Qian, Chong Zhao, Haiqiang Mai, Mingyuan Chen, Kajia Cao, Haoyuan Mo, Ling Guo, Qiuyan Chen, Linquan Tang, Wenze Qiu, Yahui Yu, Hu Liang, Xinjun Huang, Guoying Liu, Wangzhong Li, Lin Wang, Rui Sun, Xiong Zou, Shanshan Guo, Peiyu Huang, Donghua Luo, Fang Qiu, Yishan Wu, Yijun Hua, Kuiyuan Liu, Shuhui Lv, Jingjing Miao, Yanqun Xiang, Ying Sun, Xiang Guo, Xing Lv

**Affiliations:** 1 State Key Laboratory of Oncology in South China, Collaborative Innovation Center of Cancer Medicine, Guangzhou, 510060 P. R. China; 2 Department of Information, Sun Yat-Sen University Cancer Center, Guangzhou, 510060 P. R. China; 3 Department of Radiology, Sun Yat-Sen University Cancer Center, Guangzhou, 510060 P. R. China; 4 Department of Nasopharyngeal Carcinoma, Sun Yat-Sen University Cancer Center, Guangzhou, 510060 P. R. China; 5 Precision Medicine Center, Sun Yat-Sen University Cancer Center, Guangzhou, 510060 P. R. China; 6 Department of Radiotherapy, Sun Yat-Sen University Cancer Center, Guangzhou, 510060 P. R. China; 7 Guangdong Key Laboratory of Nasopharyngeal Carcinoma Diagnosis and Therapy, Guangzhou, 510060 P. R. China

**Keywords:** Nasopharyngeal malignancy, Deep learning, Differential diagnosis, Automatic segmentation

## Abstract

**Background:**

Due to the occult anatomic location of the nasopharynx and frequent presence of adenoid hyperplasia, the positive rate for malignancy identification during biopsy is low, thus leading to delayed or missed diagnosis for nasopharyngeal malignancies upon initial attempt. Here, we aimed to develop an artificial intelligence tool to detect nasopharyngeal malignancies under endoscopic examination based on deep learning.

**Methods:**

An endoscopic images-based nasopharyngeal malignancy detection model (eNPM-DM) consisting of a fully convolutional network based on the inception architecture was developed and fine-tuned using separate training and validation sets for both classification and segmentation. Briefly, a total of 28,966 qualified images were collected. Among these images, 27,536 biopsy-proven images from 7951 individuals obtained from January 1st, 2008, to December 31st, 2016, were split into the training, validation and test sets at a ratio of 7:1:2 using simple randomization. Additionally, 1430 images obtained from January 1st, 2017, to March 31st, 2017, were used as a prospective test set to compare the performance of the established model against oncologist evaluation. The dice similarity coefficient (DSC) was used to evaluate the efficiency of eNPM-DM in automatic segmentation of malignant area from the background of nasopharyngeal endoscopic images, by comparing automatic segmentation with manual segmentation performed by the experts.

**Results:**

All images were histopathologically confirmed, and included 5713 (19.7%) normal control, 19,107 (66.0%) nasopharyngeal carcinoma (NPC), 335 (1.2%) NPC and 3811 (13.2%) benign diseases. The eNPM-DM attained an overall accuracy of 88.7% (95% confidence interval (CI) 87.8%–89.5%) in detecting malignancies in the test set. In the prospective comparison phase, eNPM-DM outperformed the experts: the overall accuracy was 88.0% (95% CI 86.1%–89.6%) vs. 80.5% (95% CI 77.0%–84.0%). The eNPM-DM required less time (40 s vs. 110.0 ± 5.8 min) and exhibited encouraging performance in automatic segmentation of nasopharyngeal malignant area from the background, with an average DSC of 0.78 ± 0.24 and 0.75 ± 0.26 in the test and prospective test sets, respectively.

**Conclusions:**

The eNPM-DM outperformed oncologist evaluation in diagnostic classification of nasopharyngeal mass into benign versus malignant, and realized automatic segmentation of malignant area from the background of nasopharyngeal endoscopic images.

## Introduction

A nasopharyngeal mass is a major sign of both malignancies and benign diseases, including nasopharyngeal carcinoma (NPC), lymphoma, melanoma, minor salivary gland tumour, fibroangioma, adenoids, and cysts. Among them, NPC accounts for 83.0% of all nasopharyngeal malignancies and 4.4% of all nasopharyngeal diseases [1, 2]. Guangdong province in southern China is a highly endemic area of NPC, with an age-standardized incidence rate of 10.38/100,000 in 2013 [3].

The majority of NPC patients are diagnosed at an advanced stage, contributing to the dismal prognosis of the disease [4]. The 10-year overall survival (OS) is 50%–70% for late stage NPC patients [4–6]. Given the rapid development of imaging techniques and radiotherapy, the local control rate of early NPC patients has increased up to 95% [7]. Thus, early detection is critical for improving the OS of NPC patients.

Non-specific symptoms and an occult anatomical location are prominent causes of delayed or missed detection of nasopharyngeal malignancies. Particularly, adenoidal hypertrophy and adenoid residue in the nasopharynx is very common in adolescents and adults, respectively. Histopathological diagnosis is the gold standard for diagnosing nasopharyngeal malignancies [8]. Currently, confirmation of NPC entails a nasopharyngeal endoscopy followed by an endoscopically directed biopsy at the site of an abnormality or sampling biopsies from an endoscopically normal nasopharynx. Clinically, NPC can coexist with adenoids or is concealed in adenoid tissues [9, 10]. In this situation, repeated biopsies of the inconspicuous lesion are required; anti-tumour treatment is delayed if a repeated biopsy is needed. Abu-Ghanem et al. reported an overall negative rate of 94.2% for malignancy among patients with suspicious nasopharyngeal malignancies [11]. Endoscopic biopsies may miss small nasopharyngeal carcinomas as they are typically submucosal or located at the lateral aspect of the pharyngeal recess, presenting a substantial diagnostic challenge in the era of NPC screening.

Driven by the high performance of computing power and the advent of massive amounts of labelled data supplemented by optimized algorithms, a machine learning technique referred to as deep learning has emerged and gradually drawn the attention of investigators [12]. In particular, deep learning has recently been shown to outperform experts in visual tasks, such as playing Atari games [13] and strategic board games, such as GO [14], object recognition [15], and biomedical image identification [16–19]. To increase the diagnostic yield in distinguishing nasopharynx abnormalities, especially malignancies, via endoscopic examination, we sought to develop tools to assist in the detection of nasopharyngeal malignancies and provide biopsy guidance using a pre-trained deep learning algorithm. Sun Yat-sen University Cancer Center is a tertiary care institution located in the highly endemic area of NPC in China and has a designated department focusing exclusively on NPC. More than 3000 newly diagnosed NPC cases are treated here every year. To take advantage of deep learning methods and abundant nasopharyngeal endoscopic images in our centre, in the current study, we developed a deep learning model to detect nasopharyngeal malignancies by applying a fully convolutional network, which we termed the endoscopic images-based nasopharyngeal malignancies detection model (eNPM-DM). We investigated the diagnostic performance of eNPM-DM versus oncologists in a training set and a test set of endoscopic images of persons who underwent routine clinical screening for nasopharyngeal malignancy. We further validated eNPM-DM in a prospective set. The current study demonstrated that eNPM-DM outperformed oncologists in nasopharyngeal malignancy detection and showed encouraging performance in malignant region segmentation. This artificial intelligence platform based on eNPM-DM could provide potential benefits, such as expanded coverage of screening programmes, higher malignancy detection, and thus lower rate of repeated biopsies.

## Data and methods

### Datasets of nasopharyngeal endoscopic images

We retrospectively reviewed the clinicopathologic data and nasopharyngeal endoscopic images of persons who underwent routine clinical screening for nasopharyngeal malignancy that retrospectively collected between January 1st, 2008, and December 31st, 2016 and prospectively collected between January 1st, 2017, and March 31st, 2017 at Sun Yat-sen University Cancer Center, Guangzhou, China.

The study protocol was approved by the Ethics Committee of the authors’ affiliated institution. Consent to the study was not required because of the retrospective nature of the study. Patient data were anonymized. Furthermore, all images were de-identified and reorganized with randomized sequence disorganized in each dataset.

### Endoscopic image acquisition

All endoscopic images were acquired from each person under local anaesthesia and mucous contraction with dicaine and ephedrine. All subjects provided written informed consent for endoscopy and biopsy before anaesthesia. Images were captured using an endoscope (Model No. Storz 1232AA, KARL STORZ-Endoskope, Tuttlingen, Germany) and endoscopy capture recorder (Model No. Storz 22201011U11O). Standard white light was used during examination and image capture.

Images of patients with pathologically proven malignancy other than NPC were considered indicative of other malignancies, including lymphoma, rhabdomyosarcoma, olfactory neuroblastoma, malignant melanoma and plasmacytoma. Images of patients with precancerous and/or atypical hyperplasia, fibroangioma, leiomyoma, meningioma, minor salivary gland tumor, fungal infection, tuberculosis, chronic inflammation, adenoids and/or lymphoid hyperplasia, nasopharyngeal cyst and foreign body were considered indicative of benign diseases. Endoscopic images were eligible for analysis if they met the following criteria: (1) an image had a resolution of a minimum of 500 pixels and 70 dpi; (2) an image had a maximum size of 300 kb; (3) an image was acquired during initial diagnosis. Eligible images were randomized to the training set, the validation set and the test set at a ratio of 7:1:2. Furthermore, 1430 additional images that were independent from the training set, the validation set and the test set were used as the prospective test set to validate the established model against oncologist evaluation.

### Development and parameter tuning of the algorithm

A fully convolutional network was retrained, which could receive an input of arbitrary size and produce correspondingly sized output by deep learning [20]. The entire course of training and testing was implemented on a server with Intel^®^ Xeon^®^ CPU E5-2683/Memory: 128 GiB/GPU: GeForce GTX 1080 Ti. During training, we performed data augmentation as follows: rotation ± 30 degrees, shift ± 20%, shear 5%, zooming out/in 10% and channel shift 10%. The model was optimized for 100 epochs on the augmented training set with the initial learning rate at 0.001 and decreasing 0.1 time for each 40 epochs followed by best-model selection on the validation set.

### Evaluation of eNPM-DM in detection and segmentation of nasopharyngeal malignancies

Experts delineated the malignant area in the images of patients with biopsy-proven malignancies in each dataset. eNPM-DM was trained to distinguish malignancies from benign diseases in the training set and output the probability map for all images in each dataset. An area with a probability greater than 0.5 in an image was considered a malignant area, and the corresponding image was considered indicative of malignancy. The performance of eNPM-DM in detecting nasopharyngeal malignancies was then assessed in the test set and the prospective test set, and compared with the performance of oncologists of different seniorities in the prospective test set. They included three experts, eight resident oncologists and three interns, with greater than 5 years, greater than 1 year, or less than 3 months of working experience at the Department of Nasopharyngeal Carcinoma of Sun Yat-sen University Cancer Center, respectively. In this evaluation, we measured the standard evaluation metrics of accuracy, sensitivity, specificity, positive predictive value (PPV), negative predictive value (NPV) and time-taken for test based on images, along with the corresponding 95% confidence interval (CI) for each metric. Specificity was defined by the percentage of truly negative images divided by all images correctly identified; sensitivity was defined by the percentage of truly positive images divided by all images correctly identified. Accuracy was defined by the proportion of images correctly identified divided by all images. PPV was defined by the percentage of truly positive images divided by all images labeled as “positive”; NPV was defined by the percentage of truly negative images divided by all images labeled as “negative”. In addition, the area under curve (AUC) was calculated to assess the diagnostic efficacy of eNPM-DM in detection of nasopharyngeal malignancy using the receiver operating characteristic curve (ROC). Moreover, the combined performance of eNPM-DM and experts was assessed. An image was considered indicative of absence of malignancy if it was recognized to indicate a benign disease by either eNPM-DM or more than two experts. All analysis was performed using the Statistical Program for Social Sciences 22.0 (SPSS, Chicago, IL).

Moreover, the dice similarity coefficient (DSC) was used to evaluate the performance of eNPM-DM in automatic segmentation by measuring the overlapped ratio between expert-delineated area and eNPM-DM-defined malignant area. DSC was defined as:

$$ {\text{s}} = \frac{2|S\mathop \cap \nolimits F|}{\left| S \right| + |F|} $$

where S represents ground-truth segmentation and F stands for segmentation output.

## Results

### Demographic characteristics and disease categories of the study population

**Fig. 1:**
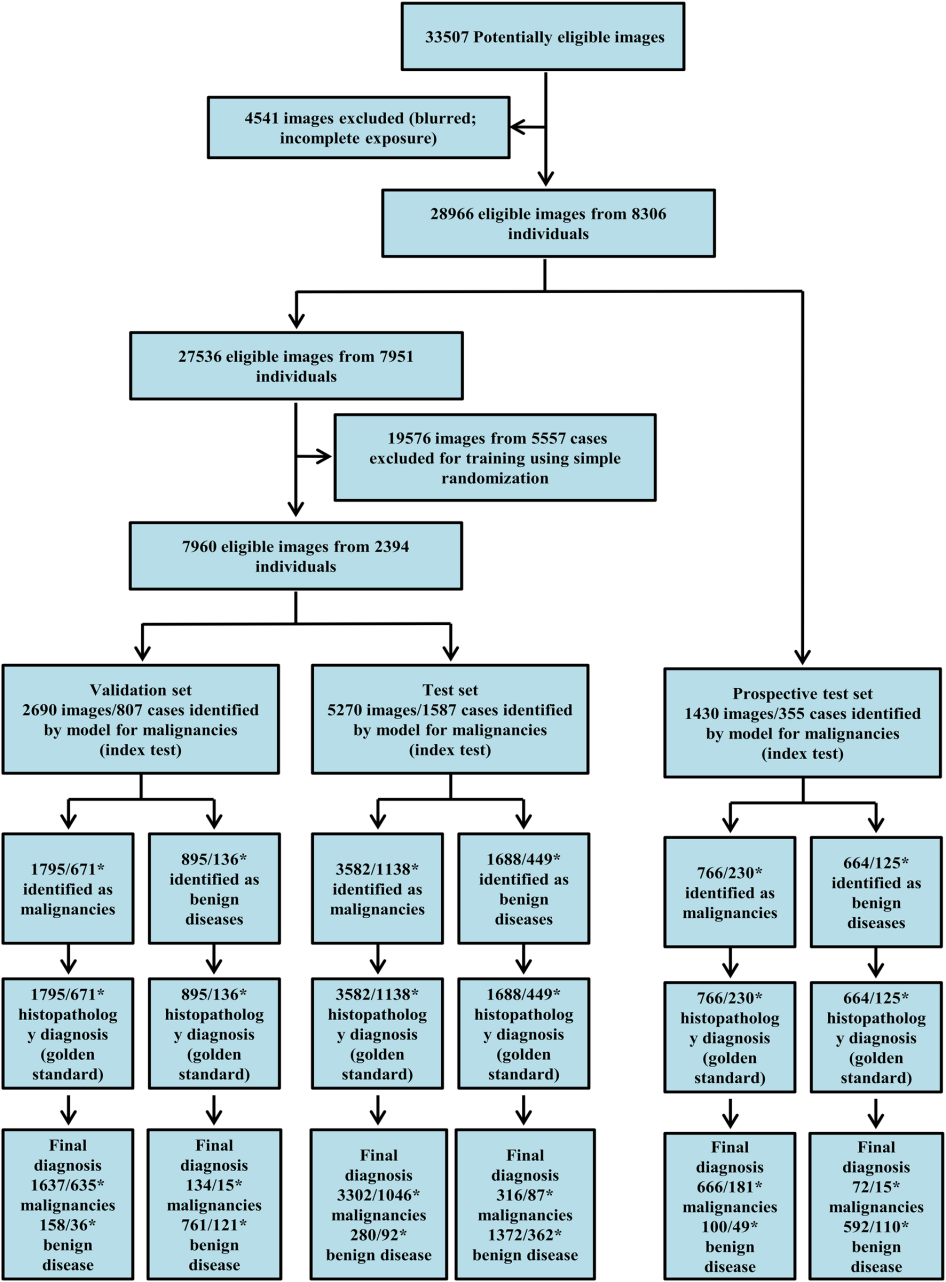
The study flowchart. *The numbers of images and cases in each subset are presented

**Fig. 2:**
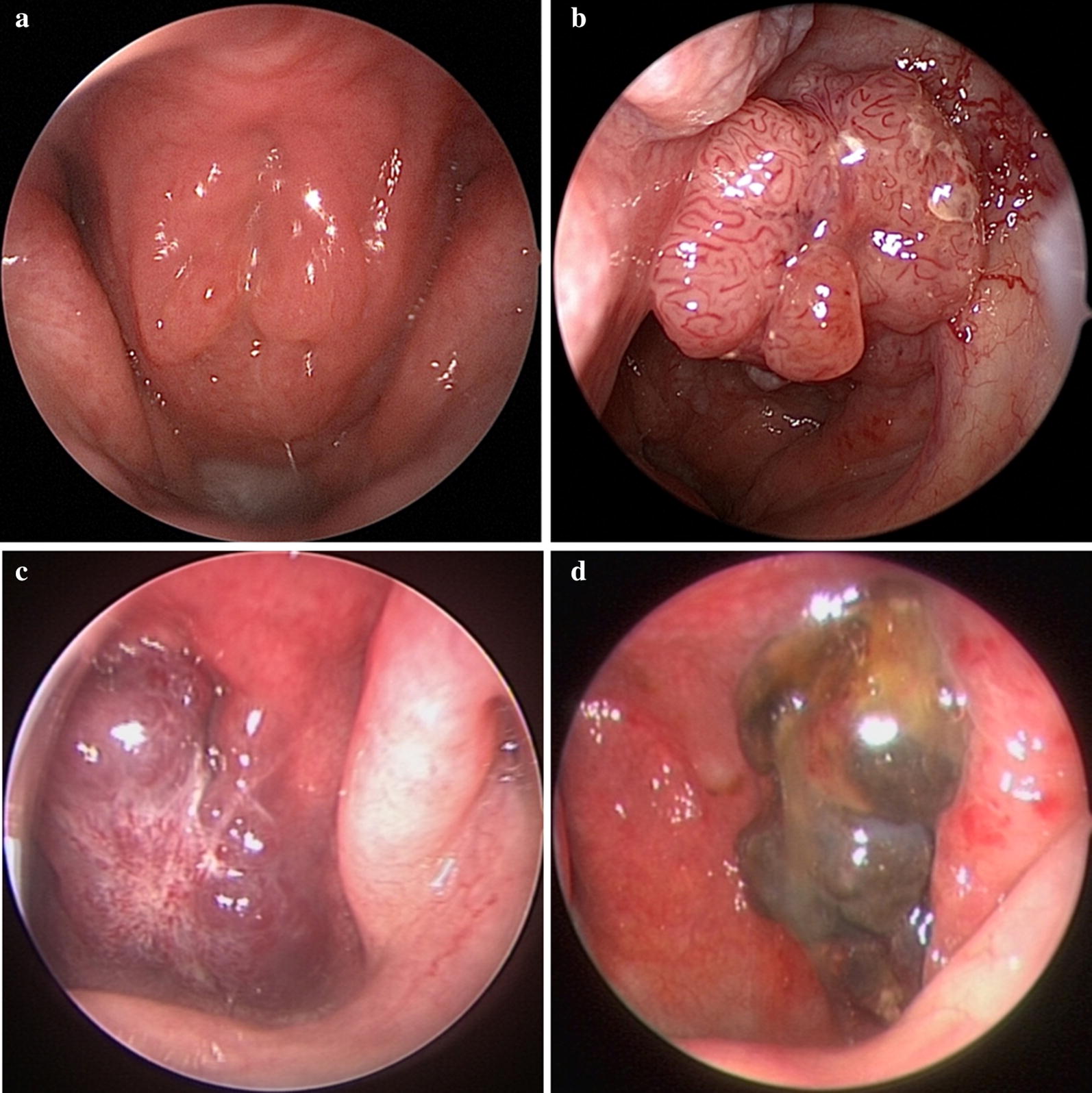
Representative images of nasopharyngeal masses. **a** normal (adenoids hyperplasia); **b** Nasopharyngeal carcinoma; **c** fibroangioma; **d** malignant melanoma

A total of 33,507 images were assessed for eligibility and 27,536 images from 7951 subjects were included for analysis. The study flowchart is shown in Fig. 1. In total, 5713 (19.7%) images came from histologically normal subjects, and 19,107 (66.0%), 335 (1.2%) and 3811 (13.2%) images came from patients with pathologically proven NPC, other malignancies and benign diseases, respectively. The training set included 19,576 images from 5557 patients, with 13,313 images from patients with biopsy-proven nasopharyngeal malignancies. The validation set was comprised of 2690 images, including 1771 images from patients with nasopharyngeal malignancies. The test set and prospective test set included 5270 images, with 3618 images from malignant cancer patients, and 1430 images, with 738 images from patients with malignancy, respectively. The demographic characteristics and disease categories of the study subjects in each dataset are shown in Table 1, and representative images of several types of nasopharyngeal masses are shown in Fig. 2.

**Table 1:** Demographic characteristics and disease categories of the study subjects in different datasets

Characteristics	All	Training set	Validation set	Test set	Prospective test set
Subjects, n	8306	5557	807	1587	355
Mean (± SD), years	45.9 ± 12.7	45.8 ± 12.7	45.9 ± 12.7	45.7 ± 12.7	47.8 ± 13.0
Sex, n(%)					
Female	2562 (30.9)	1681 (30.3)	250 (31.0)	507 (32.0)	124 (34.9)
Male	5612 (67.6)	3783 (68.1)	540 (66.9)	1058 (66.7)	231 (65.1)
N/A	132 (1.6)	93 (1.7)	17 (2.1)	22 (1.4)	0 (0.0)
Disease category, n(%)					
Normal	5713 (19.7)	3763 (19.2)	584 (21.7)	961 (18.2)	405 (28.3)
Malignancies					
NPC	19,107 (66.0)	13,061 (66.7)	1749 (65.0)	3564 (67.6)	731(51.1)
Others^a^	335 (1.2)	252 (1.3)	22 (0.8)	54 (1.0)	7 (0.4)
Benign diseases^b^	3811 (13.2)	2500 (12.8)	335 (12.5)	691 (13.1)	287(20.1)
Images, n(%)	28,966	19,576 (67.6)	2690 (9.3)	5270 (18.2)	1430 (4.9)

*N/A* not available, *NPC* nasopharyngeal carcinoma

^a^Lymphoma, rhabdomyosarcoma, olfactory neuroblastoma, malignant melanoma and plasmacytoma

^b^Precancerous/atypical hyperplasia, fibroangioma, leiomyoma, meningioma, minor salivary gland tumour, fungal infection, tuberculosis, chronic inflammation, adenoids/lymphoid hyperplasia, nasopharyngeal cyst and foreign bodies

### Diagnostic performance of eNPM-DM

**Fig. 3:**
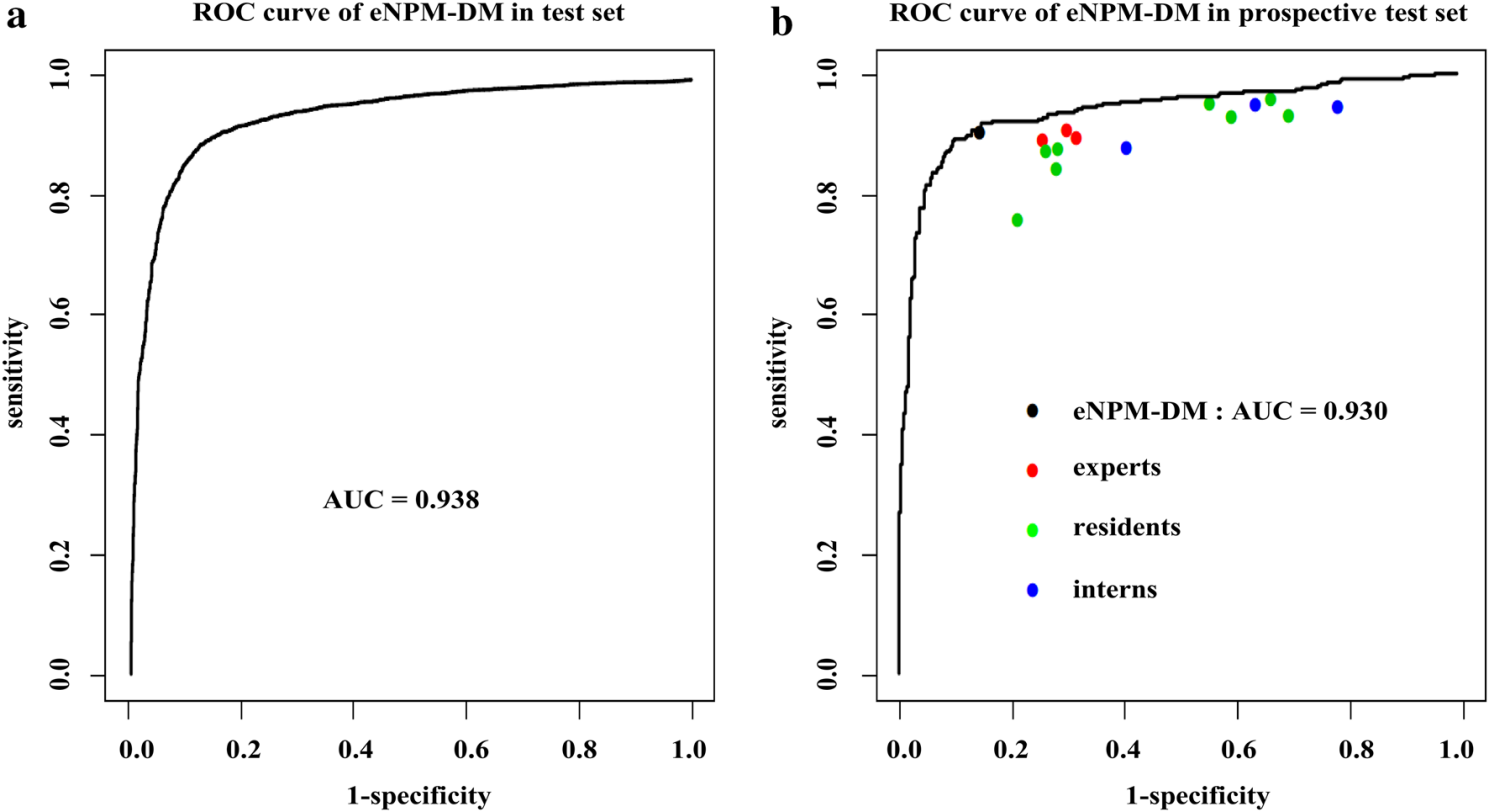
ROC for eNPM-DM in different test sets. **a** ROC of eNPM-DM in nasopharyngeal malignancy detection in the test set. **b** Comparison of the performance between eNPM-DM and oncologists with different seniorities in nasopharyngeal malignancy detection in the prospective test set. *eNPM-DM* endoscopic images-based nasopharyngeal malignancy detection model, *ROC* receiver operating characteristic curves, *AU*C area under curve

**Fig. 4:**
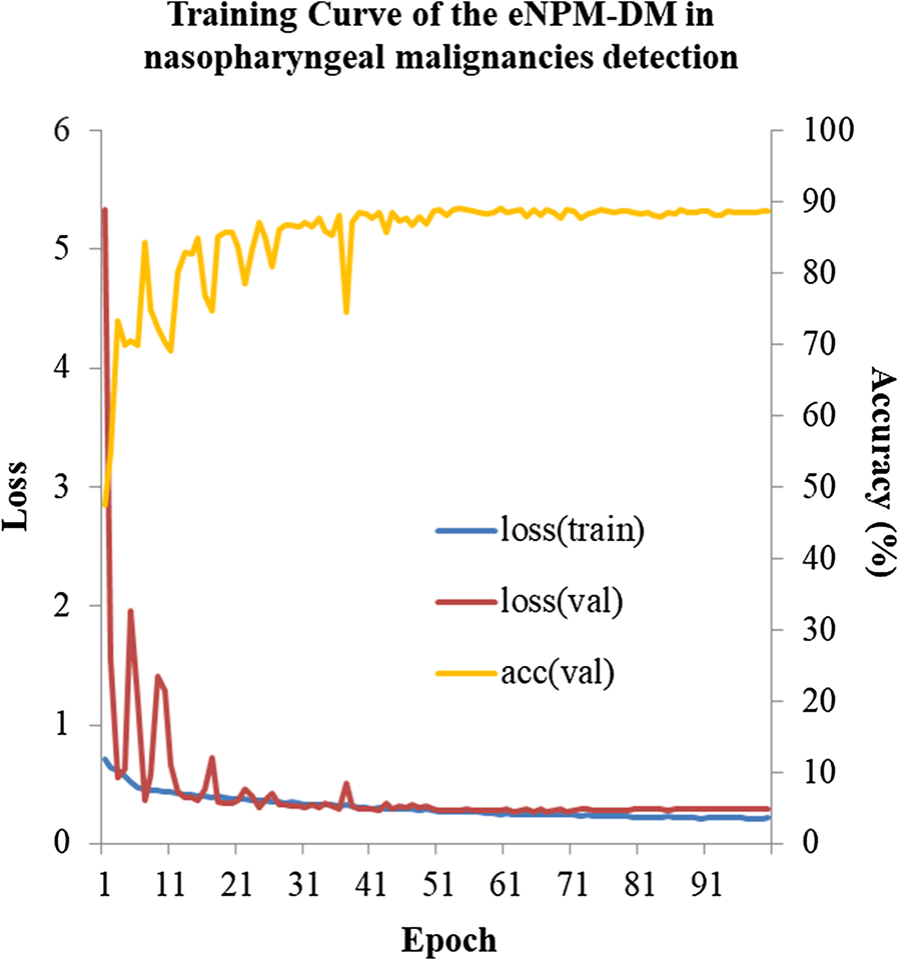
The training curve of eNPM-DM in detecting nasopharyngeal malignancies. The orange line represents the accuracy of detecting nasopharyngeal malignancies in the validation set over the course of training, with a final accuracy of 89.1% at the final epoch. The training curve was used for model selection. In this case, the best performing model at epoch 100 was used in the test and prospective test sets for final assessment. *eNPM-DM* endoscopic images-based nasopharyngeal malignancies detection model, *Val* validation

The overall accuracy of eNPM-DM for detecting malignancies in the test set was 88.7% (95% CI 87.8%–89.5%) with a sensitivity of 91.3% (95% CI 90.3%–92.2%) and a specificity of 83.1% (95% CI 81.1%–84.8%) (Table 2). We further compared the diagnostic performance of eNPM-DM with that of oncologists in nasopharyngeal malignancies in the prospective test set. eNPM-DM had an accuracy of 88.0% (95% CI 86.1%–89.6%) versus 80.5% (95% CI 77.0%–84.0%) for experts, 72.8% (95% CI 66.9%–78.6%) for residents and 66.5% (95% CI 48.0%–84.9%) for interns (Table 2). Moreover, eNPM-DM had higher specificity [85.5% (95% CI 82.7%–88.0%)] and similar sensitivity [90.2% (95% CI 87.8%–92.2%) versus experts: 70.8% (63.0%–78.6%) and 89.5% (87.4%–91.7%), respectively]. eNPM-DM also had higher PPV and NPV. eNPM-DM plus experts increased the specificity to 90.0% (95 CI 87.5%–92.1%) versus 85.5% (95% CI 82.7%–88.0%) for eNPM-DM. The AUC of eNPM-DM was 0.938 and 0.930 for nasopharyngeal malignancy in the test set and the prospective test set, respectively (Fig. 3). The training curve of eNPM-DM in nasopharyngeal malignancy detection revealed similar data loss in both the training set and the validation set, indicating no appreciable over-fitting (Fig. 4) [18].

**Table 2:** The diagnostic performance of eNPM-DM and/or oncologists in nasopharyngeal malignancy

Evaluation indicators	Test set^a^	Prospective test set^a^				
	eNPM-DM		Oncologist level			eNPM-DM plus experts
			Experts^b^	Residents^b^	Interns^b^	
Accuracy	88.7 (87.8, 89.5)	88.0 (86.1, 89.6)	80.5 ± 0.8 (77.0, 84.0)	72.8 ± 2.5 (66.9, 78.6)	66.5 ± 4.3 (48.0, 84.9)	89.0 (87.2, 90.5)
Sensitivity	91.3 (90.3, 92.2)	90.2 (87.8, 92.2)	89.5 ± 0.5 (87.4, 91.7)	88.8 ± 2.4 (83.1, 94.5)	92.2 ± 2.3 (82.1, 100.0)	87.9 (85.3, 90.2)
Specificity	83.1 (81.1, 84.8)	85.5 (82.7, 88.0)	70.8 ± 1.8 (63.0, 78.6)	55.5 ± 7.2 (38.6, 72.5)	38.9 ± 11.0 (8.5, 86.3)	90.0 (87.5, 92.1)
PPV	92.2 (91.2, 93.0)	86.9 (84.3, 89.2)	76.6 ± 1.1 (71.9, 81.3)	69.5 ± 3.1 (62.2, 76.8)	62.3 ± 3.9 (45.4, 79.2)	90.4 (87.9, 92.4)
NPV	81.3 (79.3, 83.1)	89.2 (86.5, 91.4)	86.4 ± 0.5 (84.0, 88.7)	83.2 ± 1.6 (79.4, 87.0)	82.2 ± 2.4 (71.9, 92.4)	87.5 (84.8, 90.0)
Time(min)		0.67 (~ 40 s)	110.0 ± 5.8 (85.2, 134.8)	99.3 ± 6.3 (84.3, 114.2)	106.7 ± 8.8 (68.7, 144.6)	

*eNPM-DM* endoscopic images-based nasopharyngeal malignancies detection model, *PPV* positive predictive value, *NPV* negative predictive value

^a^The numbers in parenthesis are the corresponding 95% confidence interval

^b^The performance of the oncologists is presented as mean ± standard error

Moreover, it took eNPM-DM 40 s to render an opinion, which was considerably shorter versus experts [110.0 ± 5.8 min (95% CI 85.2–134.8)].

### The performance of eNPM-DM in nasopharyngeal malignancy segmentation

**Fig. 5:**
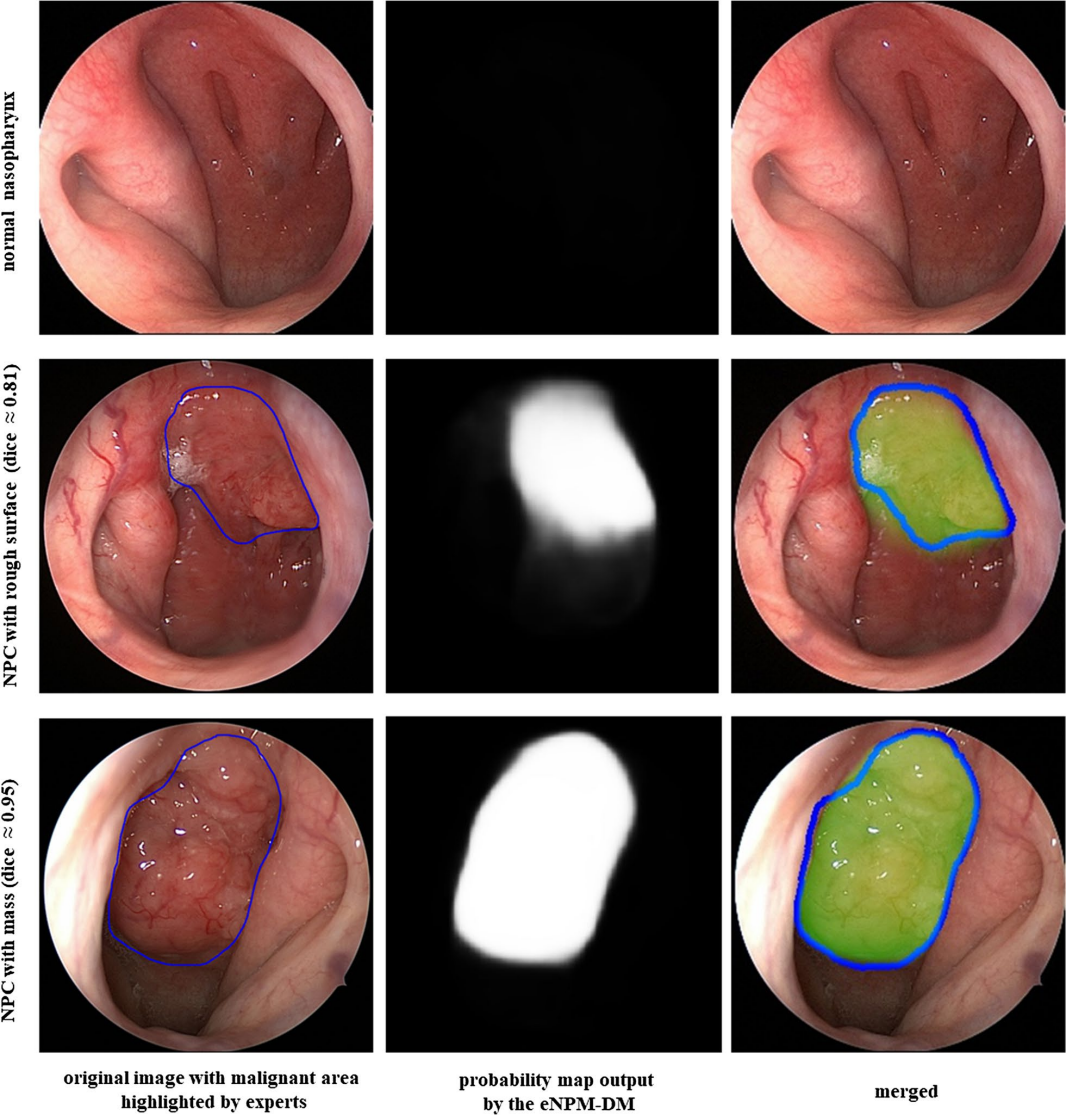
Representative images of nasopharyngeal malignancies segmentation. Images from the left to the right in each row are the original endoscopic images with or without malignant area highlighted by the experts (blue), the probability map output by eNPM-DM and the merged images of the malignant area outlined by the experts (blue) and segmented by the eNPM-DM (green). *eNPM-DM* endoscopic images-based nasopharyngeal malignancy detection model, *NPC* nasopharyngeal carcinoma

Given that a high negative rate of malignancy is the most concerning issue during biopsy due to confounded adenoid/lymphoid hyperplasia in the nasopharynx, we sought to develop a deep learning-based tool for biopsy guidance for nasopharyngeal malignancies. To address that, automatic segmentation of the malignant area from the background of nasopharyngeal endoscopic images is the most important process. No malignant area was segmented for the normal nasopharynx as the original endoscopic image was recognized as non-malignant correctly by eNPM-DM (Fig. 5). In contrast, the suspicious malignant area in an image that was recognized as malignant was segmented by eNPM-DM. As noted, eNPM-DM could recognize and segment malignant areas based on the presence of a mass or mere rough surface (Fig. 5), yielding a mean DSC of 0.78 ± 0.24 and 0.75 ± 0.26 in the test set and the prospective test set, respectively.

## Discussion

We developed an artificial intelligence model to assist physicians in the detection of nasopharyngeal malignancies and provide biopsy guidance by applying deep learning to nasopharyngeal endoscopy examination. We demonstrated that eNPM-DM was superior to oncological experts in detecting nasopharyngeal malignancies. Of note, eNPM-DM exhibited encouraging performance in nasopharyngeal malignancy segmentation.

Currently, the emerging field of machine learning, especially deep learning, has exerted significant impact on medical imaging. In general, deep learning algorithms recognize important features of images and properly giving weight to these features by modulating its inner parameters to make predictions for new data, thus accomplishing identification, classification or grading [21] and demonstrating strong processing ability and intact information retention [22], which is superior to previous machine learning methods [17]. Superiority of computer-aided diagnosis based on deep learning have recently been reported for a wide spectrum of diseases, including gastric cancer [23], diabetic retinopathy [16], cardiac arrhythmia [24], skin cancer [17] and colorectal polyp [25]. Notably, a wide variety of image types were explored in these studies, i.e., pathological slides [19, 23], electrocardiograms [24], radiological images [18, 26] and general pictures [17]. The deep learning method exhibited outstanding performance in most of the competitions between artificial intelligence and experts even though these medical images were captured by various types of equipment and presented in different forms, suggesting enormous potential of deep learning in auxiliary diagnoses. A well-trained algorithm for a specific disease can increase the accuracy of diagnosis and working efficiency of physicians, liberating them from repetitive tasks.

Recently, deep learning has been extensively used in the differential diagnosis of gastrointestinal disease in endoscopic images. Tomohiro et al. developed a convolutional neural network for detecting gastric cancer [27] and *Helicobacter pylori* infection based on endoscopic images [28]. Moreover, an artificial intelligence model was trained on endoscopic videos to differentiate diminutive adenomas from hyperplastic polyps, thus realizing real-time differential diagnosis [29]. Given that endoscopic examination is indispensable for biopsy and important for decision making in a clinical setting, developing tools for endoscopic auxiliary diagnosis can dramatically increase physicians’ working efficiency via rapid recognition and biopsy guidance, especially in patients with multi-lesions or mixed lesions [30]. Given the illusive mass caused by adenoid/lymphoid hyperplasia, it is desirable to recognize nasopharyngeal malignancies using artificial intelligence tools. However, limited studies on deep learning methods in nasopharyngeal disease differentiation have been performed based on endoscopic images to date [31]. To this end, this study has taken advantage of the abundant resource of nasopharyngeal endoscopic images at our centre and the advanced methods to develop the targeted model.

Endoscopic examination is particularly indispensable for biopsy in participants at risk of nasopharyngeal malignancies. However, currently, no additional approaches are applied to the screening of nasopharyngeal malignancies except EBV serological test [32]. Our eNPM-DM outperformed experts in distinguishing nasopharyngeal malignancies from benign diseases using far less time with encouraging sensitivity and specificity. eNPM-DM was trained and fine-tuned on numerous images that covered patients diagnosed at our centre over 8 years and exhibited encouraging performance in a shorter learning period, suggesting that eNPM-DM ‘learned’ efficiently and was highly productive.

Over diagnosis is the major cause of misdiagnosis for both eNPM-DM and oncologists, suggesting that the model might learn object recognition in the same manner as a human. For example, both could distinguish different objects based on the texture, roughness, colour, size, and even vascularity on the surface of the lesion [17]. Moreover, given increased specificity eNPM-DM versus experts, eNPM-DM may also help achieve better heath economics in NPC screening [33], simultaneously improving diagnostic accuracy and screening productivity. Furthermore, the combination of eNPM-DM and experts further increased the accuracy rate and decreased the false positive rate of NPC, identifying as many cases of malignancies as possible with minimal health expenditure in NPC screening. Accordingly, the emerging deep learning could serve as a powerful assistant in clinical practice, increasing the accuracy of screening, reducing cognitive burden on clinicians, positively impacting patients’ outcome and quality of life by fostering early intervention and reducing screening costs.

Additionally, our study offers a comprehensive method that is explicitly designed to develop a tool to segment nasopharyngeal malignancies in endoscopic images based on deep learning, which could be a promising biopsy guidance tool for nasopharyngeal malignancies, with the aim of increasing NPV of biopsy for malignancies. Here, eNPM-DM exhibited encouraging results in recognizing malignant areas in nasopharyngeal endoscopic images, which is consistent with the malignant lesion outlined by the experts. Accordingly, eNPM-DM could serve as a powerful biopsy guidance tool for resident oncologists or community physicians regardless of their limited experience in nasopharyngeal diseases.

To publicize our experience in nasopharyngeal malignancy detection and make full use of the advanced tool in clinical practice, we established an on-line platform (http://nasoai.sysucc.org.cn/). Both the patients and physicians may use this platform to assess the probability of malignancy in a certain image by uploading eligible nasopharyngeal endoscopic images to the artificial intelligence platform. If the lesion is recognized as malignant, the suggestive region for biopsy is provided.

There are limitations in this study. Given that all images were acquired from a single tertiary care centre in a highly endemic area of NPC, the diversity of nasopharyngeal diseases presented in this context might be reduced, subsequently resulting in over-fitting. However, the training curve revealed that the loss of the training was similar to that of the validation, which is indicative of a well-fit curve. In addition, NPC was the most prominent malignancy in this study, which might reduce the detection efficiency for other malignancies. However, given that NPC is the most common malignancy in the nasopharynx [1, 2] and the sensitivity of eNPM-DM in detecting nasopharyngeal malignancies was 90.2%, we believe that eNPM-DM is the most powerful auxiliary diagnosis tool in nasopharyngeal malignancy detection to date. One possible improvement could be a further increase in the spectrum of nasopharyngeal malignancies through collaboration with other centres in the future. Additionally, physical examination findings and laboratory test results, such as plasma antibody titters of EBV; and magnetic resonance imaging features of the nasopharynx and neck [9] can be taken into account during diagnosis in clinical practice. Therefore, integration of the endoscopic images, laboratory examination and radiologic images should be considered in nasopharyngeal malignancy detection based on deep learning. Similar to other deep leaning models, the exact features of eNPM-DM in malignancy detection remain unknown, and further investigation of detailed mechanisms is warranted. Particularly, since the model was trained on images, eNPM-DM could only render a diagnosis based on endoscopic images obtained in advance rather than real-time operation or video, and there is also a long and arduous way to combine eNPM-DM and the endoscopy system. Here, we manually selected 28,966 qualified images from numerous images and discarded the remaining images that are of poor quality or irrelevant. In future work, we plan to improve the performance of the model in image detection, identify the irrelevant images and evaluate image quality automatically. Finally, we plan to extend the developed deep learning image analysis framework to endoscopic image analysis and assessment in other types of cancers, such as gastric cancer, cervical cancer, and throat carcinoma.

## Conclusion

The eNPM-DM outperformed experts in detecting nasopharyngeal malignancies. Moreover, the developed model could also conduct automatic segmentation of malignant area from the confusing background of nasopharyngeal endoscopic images efficiently, showing promising prospects in biopsy guidance for nasopharyngeal malignancies.

## Abbreviations

eNPM-DM: endoscopic images-based nasopharyngeal malignancy detection model
NPC: nasopharyngeal carcinoma
